# Supplementary material for: Optimization of Sensory Properties of Cold Brew Coffee Produced by Reduced Pressure Cycles and Its Physicochemical Characteristics
Source: Molecules. 2022 May 6;27(9):2971. doi: 10.3390/molecules27092971 (PMC9104833; doi:10.3390/molecules27092971)
Supplement: Supplementary file 1 [file molecules-27-02971-s001.zip › molecules-1697906-supplementary.pdf]

molecules

MDPI

Supplementary Materials

Table S1. Comparison of caffeine and phenolic content for 7 and 13 vacuum cycles (coffee to water ratio 1/9).

Vacuum cycles	Pressure (mbar)	Vacuum time (s)	Caffeine (mg/g coffee)	Phenols (mg gallic acid/g coffee)
7	205	300	9.61±0.324^{A}	14.33±0.253^{A}
13	205	300	10.02±0.118^{A}	15.12±0.417^{A}

Table S2. Comparison of lipids for the examined grinding degrees (determination by Folch method).

Grinding degree (microns)	Lipids (mg/ g coffee extract)
600-700	2.8±0.05^{C}
700-800	4.2±0.04^{B}
800-1000	4.3±0.04^{B}
>1000	4.6±0.08^{A}

Table S3. L*, a*, b* values for varying vacuum cycles (constant pressure).

Vacuum cycles	L*	a*	b*
2	36.9±0.3^{BC}	23.3±0.6^{C}	56.9±2.3^{C}
5	35.9±0.2^{C}	24.0±0.2^{C}	56.4±1.3^{C}
7	33.9±0.8^{D}	24.5±0.4^{C}	54.3±1.0^{C}
10	43.7±0.04^{A}	30.1±0.3^{A}	72.4±0.1^{A}
13	29.7±0.8^{E}	28.0±0.6^{B}	49.7±1.0^{D}
16	37.9±0.4^{B}	28.8±0.8^{AB}	62.6±1.1^{B}

Table S4. L*, a*, b* values for varying values of pressure (2 and 7 vacuum cycles).

	2 cycles			7 cycles		
Pressure (mbar)	L*	a*	b*	L*	a*	b*
20	45.0±0.2^{B}	25.2±0.7^{AB}	74.2±0.3^{B}	45.9±0.4^{A}	28.8±0.5^{A}	73.4±1.5^{A}
50	39.3±0.4^{C}	25.9±1.1^{A}	65.2±0.6^{C}	38.2±0.6^{B}	28.0±0.4^{AB}	63.6±1.1^{B}
100	50.0±0.1^{A}	24.6±0.3^{AB}	80.6±0.4^{A}	35.3±1.6^{C}	27.8±0.8^{AB}	58.7±2.9^{C}
300	49.4±0.5^{A}	26.7±0.3^{A}	79.5±0.7^{A}	40.2±0.5^{B}	26.6±1.2^{B}	65.4±0.2^{B}
500	33.9±0.5^{D}	23.2±0.4^{BC}	54.1±1.7^{D}	44.3±0.3^{A}	23.2±0.4^{C}	54.1±1.7^{C}
700	40.2±1.1^{C}	21.7±0.6^{C}	64.5±1.3^{C}	44.7±0.3^{A}	27.8±0.2^{AB}	74.0±0.4^{A}

Table S5. Statistical analysis of the effect of cycles at constant pressure on sensory properties of cold brew coffee (corresponds to Figure 2) (avg±SD, n=6).

Cycles	Aroma	Flavor	Acidity	Aftertaste	Body	Balance
2	7.92±0.27^{A}	7.26±0.12^{A}	8.26±0.23^{A}	6.87±0.24^{A}	7.97±0.23^{A}	7.03±0.18^{A}
5	6.25±0.27^{C}	7.10±0.15^{A}	7.33±0.29^{B}	7.03±0.19^{A}	6.33±0.22^{C}	7.03±0.12^{A}
7	6.28±0.18^{C}	7.03±0.15^{A}	7.33±0.25^{B}	6.97±0.19^{A}	7.03±0.21^{B}	7.00±0.17^{A}
10	6.28±0.19^{C}	6.49±0.28^{B}	7.53±0.29^{B}	6.33±0.21^{B}	6.45±0.23^{C}	6.25±0.37^{B}
13	6.93±0.12^{B}	6.40±0.28^{B}	6.37±0.18^{C}	6.33±0.20^{B}	6.45±0.23^{C}	6.43±0.19^{BC}
16	6.77±0.23^{B}	6.23±0.23^{B}	6.28±0.23^{C}	6.00±0.14^{B}	6.40±0.21^{C}	6.02±0.19^{C}

Means that do not share a letter are significantly different.

Table S6. Statistical analysis of the effect of pressure for 2 cycles on sensory properties of cold brew coffee (corresponds to Figure 3a) (avg±SD, n=6).

Pressure (mbar)	Aroma	Flavor	Acidity	Aftertaste	Body	Balance
20	6.02±0.15^{C}	6.13±0.12^{C}	6.00±0.23^{B}	6.03±0.23^{B}	6.03±0.26^{C}	6.03±0.19^{B}
50	6.08±0.21^{BC}	6.15±0.24^{C}	6.00±0.17^{B}	6.00±0.14^{B}	6.07±0.22^{C}	6.02±0.16^{B}
100	6.47±0.24^{A}	7.13±0.20^{A}	7.53±0.23^{A}	6.25±0.23^{B}	6.33±0.28^{BC}	7.02±0.28^{A}
300	6.17±0.19^{ABC}	6.67±0.28^{B}	6.22±0.25^{B}	7.25±0.15^{A}	7.03±0.21^{A}	6.97±0.22^{A}
500	6.41±0.22^{AB}	6.67±0.15^{B}	6.22±0.21^{B}	6.38±0.23^{B}	6.50±0.21^{B}	6.98±0.36^{A}
700	6.08±0.26^{BC}	6.33±0.19^{BC}	6.10±0.12^{B}	6.03±0.31^{B}	6.00±0.22^{C}	5.98±0.25^{B}

Means that do not share a letter are significantly different.

Table S7. Statistical analysis of the effect of pressure for 7 cycles on sensory properties of cold brew coffee (corresponds to Figure 3b) (avg±SD, n=6).

**Pressure (mbar)**	**Aroma**	**Flavor**	**Acidity**	**Aftertaste**	**Body**	**Balance**
20	6.42 ± 0.12^{A}	6.58 ± 0.12^{A}	6.42 ± 0.12^{B}	6.08 ± 0.12^{B}	6.67 ± 0.12^{A}	6.75 ± 0.20^{A}
50	6.41 ± 0.12^{A}	6.67 ± 0.12^{A}	7.25 ± 0.20^{A}	7.08 ± 0.12^{A}	6.17 ± 0.12^{B}	6.75 ± 0.13^{A}
100	6.45 ± 0.12^{A}	6.68 ± 0.12^{A}	7.17 ± 0.24^{A}	6.75 ± 0.20^{A}	6.17 ± 0.12^{B}	6.58 ± 0.11^{B}
300	6.01 ± 0.11^{B}	6.12 ± 0.10^{B}	6.03 ± 0.05^{B}	6.05 ± 0.09^{B}	6.08 ± 0.12^{B}	6.10 ± 0.10^{C}
500	6.08 ± 0.12^{AB}	6.15 ± 0.11^{B}	6.03 ± 0.12^{B}	6.00 ± 0.14^{B}	6.13 ± 0.09^{B}	6.04 ± 0.04^{C}
700	6.18 ± 0.13^{AB}	6.08 ± 0.12^{B}	6.08 ± 0.12^{B}	6.08 ± 0.12^{B}	6.07 ± 0.09^{B}	6.01 ± 0.10^{C}

Means that do not share a letter are significantly different.
